# Principles of Fracture Healing and Fixation: A Literature Review

**DOI:** 10.7759/cureus.76250

**Published:** 2024-12-23

**Authors:** Mohammad Waseem Beeharry, Belal Ahmad

**Affiliations:** 1 Trauma and Orthopaedics, Royal Surrey NHS Foundation Trust, Guildford, GBR

**Keywords:** fixation constructs, fracture fixation, fracture healing, principles of fracture fixation, stages of fracture healing

## Abstract

Bone healing is a complex, dynamic process involving a series of well-coordinated stages, influenced by both mechanical and biological factors. The skeletal system, composed of inorganic (36%), organic (36%), and water (28%) components by volume, plays a crucial role in maintaining structural integrity and mineral homeostasis. Bone is classified into two main types based on microstructure: lamellar and woven bone, with lamellar bone being stronger and more durable. Factors such as inflammation, the periosteum, vascularity, and infection significantly impact healing outcomes. Moreover, fracture fixation is fundamental to optimal healing, guided by principles of anatomical reduction, stable fixation, blood supply preservation, and early mobilisation. Perren’s strain theory emphasises the importance of strain at the fracture site, which can determine whether primary or secondary healing occurs. Rigid fixation provides an environment which promotes primary bone healing, while flexible fixation promotes secondary healing through controlled motion. Internal and external fixation methods, including plates, screws, and intramedullary nails, offer varying degrees of stability, supporting bone healing. Overall, optimal fracture fixation, combined with an understanding of bone biology, enhances healing and functional recovery.

## Introduction and background

Effective fracture management necessitates a comprehensive understanding of bone composition, types, and the complex mechanisms involved in bone healing. Bone is a dynamic, multi-component tissue that not only provides structural support and protection to vital organs but also plays a crucial role in maintaining mineral homeostasis within the body. The healing process is a highly intricate and coordinated sequence of events, encompassing inflammation, cellular recruitment, and remodelling, and depends on a balance of biological and mechanical factors. Factors such as smoking, infection, and compromised vascularisation can severely hinder this process and, therefore, negatively impact both short- and long-term outcomes in fracture recovery. Modern principles of fracture fixation, underpinned by biomechanical theories, aim to stabilise fractures and optimise healing. This literature review delves into the complex, multi-phase process of fracture healing, with an emphasis on fixation techniques for successful skeletal repair.

## Review

Principles of fracture healing

Composition of Bone

The skeletal system comprises 206 bones, making up approximately 15% of an adult’s total body weight [1]. Bone is a dynamic tissue which continuously undergoes remodelling. It is composed of an inorganic component (36%), organic component (36%) and water (28%) by volume [2]. In addition to these, bone contains various cellular components, including osteocytes, osteoblasts, and osteoclasts, each playing a crucial role in bone formation and maintenance.

The inorganic component is primarily composed of thin plates of hydroxyapatite crystals which contain high levels of calcium and phosphate. The function of the inorganic component is to provide strength and hardness to bone and, as it houses the body’s mineral reserves of calcium, phosphorus, sodium and magnesium, it has a crucial role in maintaining mineral homeostasis [3].

The organic component is predominantly composed of collagen, accounting for approximately 90% of its composition, with type 1 collagen being the primary form. The remaining 10% consists of non-collagenous proteins, contributing to the overall protein content of bone, and plays an important role in various biological processes, including mineralisation, bone remodelling, cell signalling, and regulation of bone cell activity [2]. Collagen provides tensile strength and flexibility to the bone, and with its fibrous structure, it forms a scaffold which allows the deposition of hydroxyapatite crystals which gives bone its hardness [4].

The intricate composition of bone, driven by the dynamic collaboration between its organic and inorganic components, is essential for its crucial role in the body. This enables bones to serve a wide variety of vital functions such as providing structural support to the body, shielding internal organs from injury, facilitating movement by serving as anchor points for muscles, acting as a reservoir for important minerals such as calcium, and housing the bone marrow, where hematopoiesis occurs [5-7].

Bone Characteristics

Microscopically, there are two main types of bone: lamellar bone and woven bone. The mature, well-organised form of bone tissue is called lamellar bone. In lamellar bone, the collagen fibres are arranged into thin layers called lamellae in arch-like patterns to maximise bone strength. This organisation enables the tissue to achieve the highest collagen density per unit volume. The distinctive structure of compact bone is formed by the stacking of these lamellae in concentric circles around Haversian canals, which contain nerves and blood vessels. Lamellar bone is further divided into cortical bone and trabecular bone.

The other type of bone based on microstructure is woven bone. Woven bone is a type of bone tissue that forms when the bone is being formed very rapidly such as during development, in the early stages of fracture healing, or in certain tumours and metabolic bone diseases. The collagen fibres form randomly orientated bundles and are loosely packed [8]. Therefore, lamellar bone, with its organised collagen pattern, is strong and durable, while woven bone, produced in high-turnover states such as in Paget’s disease, is weaker due to its lack of organisation and structure [9].

Macroscopically, bones are classified into four types: long, short, flat, and irregular. Long bones, such as the femur, tibia, and humerus, develop through both endochondral as well as membranous ossification. On the other hand, flat bones, such as the skull, scapula, mandible, and ribs, develop by membranous ossification only.

The skeleton comprises 80% cortical and 20% trabecular bone, with varying ratios of cortical to trabecular bone at different sites and therefore affecting bone density and strength [8]. For example, the ratio of cortical to trabecular bone is 50:50 in the femoral head, 95:5 in the radial diaphysis, and 25:75 in the vertebras.

Cortical bone is dense and surrounds the marrow, containing cylindrical osteons, while trabecular bone has a honeycomb network, ideal for metabolic activity. Both cortical and trabecular bone are composed of osteons. Cortical bone has an outer surface called the periosteal surface and an inner surface known as the endosteal surface. This periosteal surface is crucial for bone growth and fracture repair as this is where cortical bone thickens as we age. In contrast, bone resorption typically exceeds formation on the endosteal surface, causing the marrow space to expand with ageing [8].

Bone growth and maintenance involve longitudinal growth, modelling, and remodelling processes that respond to developmental and physiological forces. During childhood and adolescence, longitudinal growth occurs at the growth plates where cartilage rapidly grows in the epiphyseal and metaphyseal regions of long bones, forming primary bone tissue.

Bone Modelling

Bone modelling is a dynamic and adaptive process by which bones adapt their shape in response to physiological or mechanical forces, gradually reshaping the skeleton to withstand and accommodate stresses. This occurs through the independent actions of osteoblasts and osteoclasts, which add or remove bone at specific surfaces. For instance, as individuals age, bones widen through periosteal apposition (bone formation on the outer surface) and endosteal resorption (bone removal from the inner surface), a natural adjustment that accommodates changing mechanical demands. Wolff's law highlights that long bones remodel their structure to align with mechanical stresses. Unlike remodelling, modelling is less frequent in adults and is not tightly coupled. Conditions such as hypoparathyroidism, renal osteodystrophy, or anabolic treatments can increase modelling activity [8,10].

Bone Remodelling

Bone remodelling involves the continuous renewal of bone to maintain strength and mineral balance. This process replaces old bone with new bone through a tightly coupled sequence involving osteoclast-driven resorption, followed by osteoblast-driven formation. Remodelling prevents microdamage accumulation and operates throughout life, beginning before birth. The process accelerates during peri-menopause and early post-menopause but slows with further aging, while still exceeding premenopausal rates. In men, remodelling increases modestly with age.

The bone remodelling cycle is a highly orchestrated process that unfolds in four distinct phases: activation, resorption, reversal, and formation. While some remodelling occurs spontaneously, it is often directed towards regions in need of repair or adaptation. The end result ultimately gives rise to the formation of a new osteon, a fundamental unit of bone structure. The remodelling of both cortical and trabecular bones follows similar principles, albeit tailored to the unique architectural characteristics and mechanical demands of their respective tissues. Over time, endosteal and trabecular resorption outpaces periosteal formation, leading to cortical and trabecular thinning with age [8,10].

Bone modelling allows bones to reshape in response to mechanical forces by osteoblasts and osteoclasts working independently, resulting in bone widening with age, consistent with Wolff's law [8]. In adults, remodelling prevails, maintaining bone strength and mineral balance. This remodelling cycle includes phases of bone resorption and formation. Osteoclasts play a key role, creating resorption cavities, known as Howship’s lacunae, in trabecular bone and resorbing cortical bone through a cone-cutting mechanism [10]. The remodelling cycle continues as osteoblasts deposit a new matrix, which mineralises to become bone. This complex process prevents microdamage build-up and is regulated by various signals, with osteocytes and bone-lining cells forming an extensive network that responds to mechanical stress and hormonal changes.

Stages of Fracture Healing

Fracture healing is a complex process involving anabolic and catabolic phases, with key stages such as hematoma formation, granulation tissue formation, callus formation, and bone remodelling [11]. Initially, a hematoma forms at the fracture site, followed by the recruitment of mesenchymal stem cells (MSCs) as well as inflammatory cells. These cells release cytokines, triggering inflammation and tissue repair. Granulation tissue forms within two weeks, offering provisional stability, and MSCs differentiate into chondrocytes, forming a fibrocartilaginous callus. As healing progresses, the cartilage is replaced by woven bone through endochondral ossification.

Bony callus formation occurs when fibroblasts, influenced by bone morphogenetic proteins (BMPs), differentiate into osteoblasts, laying down bone matrix. The cartilaginous callus is resorbed, calcified, and replaced with a hard, immature bone. Bone remodelling then begins, lasting months to years, and involves osteoclasts resorbing bone and osteoblasts forming new bone [12]. This remodelling restores bone structure, with compact bone replacing the callus centre, and lamellar bone replacing the edges. The entire remodelling process is meticulously regulated by complex signalling pathways, including BMPs, which ensure the proper differentiation of cells and the progressive maturation of the bone. Additionally, mechanical stresses, as outlined by Wolff’s law, play a vital role in guiding the bone’s final shape and density, aligning the bone’s architecture to withstand the specific loads it will encounter. The remodelled bone reflects coordinated actions of osteoclast and osteoblast. This includes the formation of Howship’s lacunae, the cavities created by osteoclasts during bone resorption, and the cone-cutting process, where osteoclasts carve out channels to allow for new bone formation [11].

How do fractures heal?

Inflammation

Macrophages significantly influence fracture healing by promoting bone remodelling and repair [13]. Tissue-resident macrophages, closely associated with osteoblasts, contribute to normal bone healing, and enhancing macrophage influx accelerates recovery. Conversely, blocking macrophage influx slows healing [14,15]. However, inflammation can hinder bone healing, particularly in cases of sustained inflammation or in conditions such as diabetes, smoking, and ageing. Studies show that juvenile bone marrow can stimulate faster healing in older animals, indicating that age impacts the inflammatory response to fractures [16]. While macrophages generally support healing in younger individuals, they may delay recovery in older ones due to changes in their ability to shift from a pro-inflammatory to an anti-inflammatory state. This age-dependent effect highlights how inflammation dynamics vary across life stages, influencing fracture outcomes.

While excessive inflammation can hinder healing, insufficient inflammation may also delay bone repair and increase the incidence of delayed osseous healing. A highly debated topic in recent years is the use of non-steroidal anti-inflammatory drugs (NSAIDs) and their effects on fracture healing. These drugs, commonly prescribed post-surgery for their anti-inflammatory and pain-relieving effects, have been linked to impaired bone healing, increased rates of non-union, and reduced bone strength [17]. Although the exact mechanisms by which altered inflammation disrupts healing remain controversial, maintaining a carefully regulated inflammatory response is thought to be essential for effective bone repair.

Periosteum

The periosteum plays a central role in fracture healing, as it houses the primary stem and progenitor cells necessary for regenerating bone and cartilage. Earlier beliefs held that MSCs from systemic circulation contributed to healing by migrating to injury sites; however, research indicates these cells do not significantly contribute to bone and cartilage formation [18]. Instead, lineage tracing studies demonstrate that the periosteum and endosteum provide the majority of cells for fracture repair [19]. The periosteum generates both bone and cartilage, while the endosteum primarily forms bone [16]. Additionally, local periosteal cells proliferate and differentiate into chondrocytes and osteoblasts near their origin, underscoring the periosteum's essential role. Although other cell types may also assist, these findings highlight the periosteum as the primary tissue source for stem cells critical to successful fracture healing.

Vasculature

Bone fracture healing is intricately dependent on both vascularisation and oxygen levels, two critical factors that directly influence the healing process. A strong angiogenic response supports bone formation, provides vital nutrients, and delivers systemically derived cells to the fracture site [20]. Blood vessels also contribute directly, as their endothelial cells produce BMPs, enhancing bone regeneration [21]. Oxygen, too, is a crucial player, with its effects being complex and multifaceted. Hypoxia can support chondrogenesis, the formation of cartilage, while higher oxygen levels promote osteogenesis, the formation of bone. Experiments show that fractures in hypoxic conditions have reduced angiogenesis and slower healing, while hyperoxia can hinder healing by generating harmful radicals, although it aids ischemic cases by stimulating collateral vessel formation [22,23]. Monitoring oxygen levels at the fracture site reveals that low oxygen areas initially encourage cartilage growth, while higher oxygen supports bone formation. This highlights vascular and oxygen management as promising therapeutic targets, although clinical application remains limited.

Infection

Infection is one of the most prominent factors that impede fracture healing, profoundly disrupting the body’s natural ability to regenerate and repair bone tissue. When bacteria invade the fracture site, they can disrupt the healing process, leading to delayed union, non-union, and poor patient outcomes [24]. Managing fractures complicated by infection presents a significant challenge, as it demands a delicate balance between ensuring proper bone stabilisation and effectively controlling the infection. Orthopaedic hardware, essential for fracture reduction and stability, can increase the risk of infection when bacteria enter the body. As a foreign object, the implant provides a surface that promotes bacterial colonisation and biofilm formation, making infections more difficult to treat with systemic antibiotics. The presence of the implant creates an environment where bacteria are more likely to thrive, complicating the body’s ability to fight off the infection.

Managing fracture-related infections presents a challenging dilemma, as clinicians must decide whether to prioritise eradicating the infection or promoting the healing of the fracture. One approach is to prioritise clearing the infection by removing fixation devices to eliminate biofilm sites, extended courses of antibiotics, and multiple surgical debridement. However, this approach may compromise fracture reduction and necessitate the use of less stable stabilisation methods such as external fixation. The alternative would be to prioritise achieving optimal fracture reduction, even if it means maintaining implant surfaces that may create an environment where the infection could continue to thrive. While this approach may enhance the alignment and healing of the bone, it carries the significant risk of allowing the infection to persist, potentially complicating recovery and prolonging the treatment process [24].

Osteomyelitis, a severe and often debilitating bone infection, significantly impairs the natural process of bone healing, leading to potentially catastrophic consequences such as permanent functional loss or, in the most extreme cases, amputation. This infection not only jeopardises the integrity of the bone but also complicates recovery, making it a critical concern in trauma care. Infection rates vary widely, with closed fractures experiencing rates as low as 1-2%, while those involving open fractures can soar to alarming levels of up to 30% [25]. It hinders callus formation by promoting fibrous tissue, weakens mechanical stability, and affects overall healing. Symptoms of osteomyelitis often include persistent pain in the affected bone, accompanied by a high fever and general fatigue or lethargy. The area may become visibly inflamed, with redness and warmth, and there is typically significant tenderness to the touch [26].

Smoking

Tobacco smoke harms the immune system, increasing infection risks, especially in surgical wounds. Key substances, nicotine and carbon monoxide (CO), worsen tissue oxygenation: CO blocks haemoglobin binding sites, while nicotine triggers vasoconstriction. Other smoke components damage erythrocytes and endothelium, promoting wound hypoxia and bacterial growth, hindering healing [27].

Evidence suggests that smoking delays bone healing post-fracture or surgery [28,29]. Smokers, particularly with open fractures, experience prolonged healing times, higher rates of non-union, and increased infection risks [30]. Studies show that nicotine reduces blood flow, impairing tissue oxygenation and bone angiogenesis [31]. Clinical data and animal studies indicate that smoking causes tissue hypoxia and disrupts critical growth factors such as VEGF (vascular endothelial growth factor) and TGF-β (transforming growth factor beta), which are needed for bone repair [32,33]. Although a direct causative link between smoking and delayed union is not fully established, associations and biological mechanisms are well-supported.

Research shows that smoking increases non-union rates in fracture healing across limbs, regardless of treatment method [34]. While cessation is advised before surgery, smokers face higher infection risks and reduced MSCs [35]. Emerging treatments include local transplantation of bone marrow cells to protect grafts and MSC-based therapies to enhance non-union repair [36].

Principles of fracture fixation

Achieving optimal fracture healing requires a thorough understanding of all the factors that directly or indirectly influences the healing process. The AO has outlined four fundamental principles for optimal fracture healing: reduction to restore anatomy, stable fixation to provide absolute or relative stability of the fracture, maintaining of adequate blood supply, and restoration of function through early mobilisation [37].

**Figure 1:**
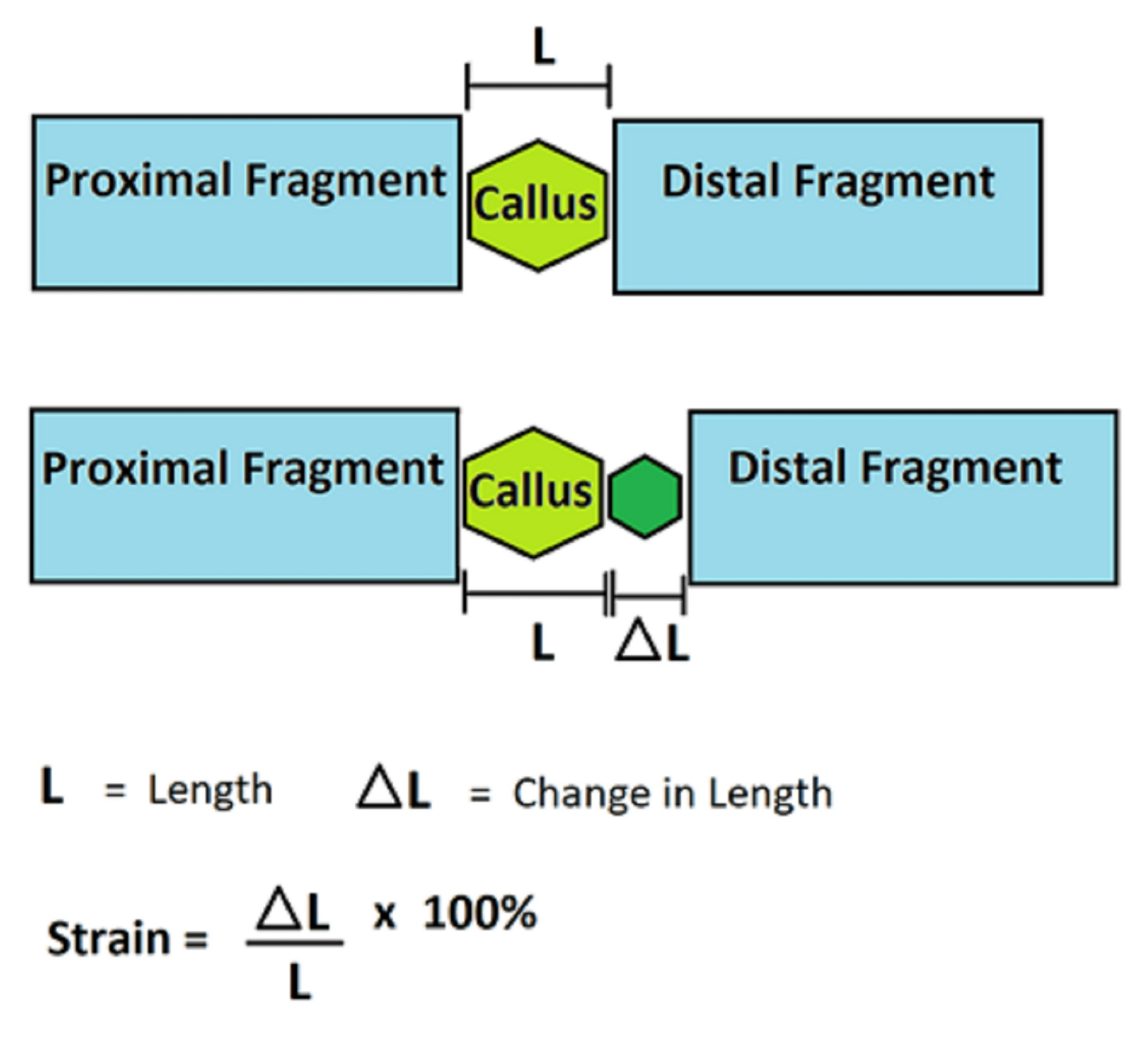
Perren's strain theory Figure 1 is an original work of the authors

The evolution of our current understanding of the manner in which mechanical factors influence the fracture healing process can be best explained through the globally renowned Perren's strain theory. This theory revolves around the concept of strain, described as the amount of movement occurring at a fracture site when a given mechanical force (such as body weight or muscle activity) is applied. It is quantified by the change in length of one bone fragment relative to the other over the original length (delta L / L) [38]. Perren's strain theory is illustrated in Figure 1.

In the context of orthopaedic trauma, the type of fixation used (e.g., rigid vs. flexible) determines the amount of strain at the fracture site. Hence, optimising the strain environment and the methods used for their fixation play a crucial role in determining the healing process. This is key as it dictates the type of bone healing which ensues by one of the two main mechanisms, either primary bone healing (or direct bone healing) or secondary bone healing.

A low mechanical strain environment (<2%) at a fracture site is achieved by an absolute stability construct, and this promotes bone formation [39]. Absolute stability refers to the optimal scenario in which the fractured bone fragments are anatomically reduced and fixed in such a way that it prevents any fracture micromotion between fragments under typical physiological stresses. This is typically achieved through the use of rigid fixation techniques such as plates and screws with lag techniques.

This lack of movement between fragments promotes primary bone healing, in which the bone heals through direct osteonal bridging (intramembranous ossification). This eventually matures into lamellar bone consisting of highly organised sheets of mineralised osteoid which results in fracture healing without periosteal callus formation.

In contrast, a moderate mechanical strain (between 2% and 10%) supports secondary bone healing as a result of relative stability. Relative stability allows for controlled movement between the bone fragments when under physiological load. Although this does not achieve the same level of anatomical reduction as absolute stability, the emphasis is on the correction of alignment, length, and rotation. The main objective is to limit motion to a degree that facilitates healing, accepting some micro-motion in the process. This type of stabilisation can be commonly attained through immobilisation in a plaster or surgically through external fixation, intramedullary nails (IMNs), or other forms of fixation that are less rigid such as bridge plating. As a result of this, secondary bone healing takes place, whereby endochondral ossification results in the formation of a callus, which serves as a link between the fragments [40].

Objectives of fracture fixation and varieties of fixation constructs

The main aims of fracture fixation are the restoration of anatomical alignment with a view to preserving the surrounding soft tissues. In addition to restoring anatomy, a stable fixation should be sought, as this not only supports the bone but also prevents any to little movement at the fracture site during healing, depending on the construct used. This results in accelerated recovery, with more predictable and faster healing, ultimately leading to improved outcomes for the patient in terms of functional recovery and allowing early mobilisation.

There are primarily two main types of fracture fixation used in clinical practice: internal fixation and external fixation. Any internal and external fixation methods that permit controlled interfragmentary movement during functional weight-bearing are considered flexible fixation. Conversely, those that use compression mechanisms are classified as rigid fixation [41].

Internal fixation devices can be broadly categorised into different types of hardware constructs such as wires, plates and screws, pins, and IMNs or rods for fracture fixation at different skeletal locations. These devices provide immediate mechanical support to the fracture site and are typically made from stainless steel and titanium alloys, both of which are known for their durability and strength. Stainless steel, with a high Young's modulus of approximately 200 gigapascals (GPa), offers adequate strength for plate and screw fixation constructs. Although stainless steel is resistant to corrosion, its corrosion resistance is not ideal. To enhance this performance, titanium and its alloys, which have a Young's modulus of approximately 110 GPa, have been introduced for use in internal fracture fixation to improve this performance [42,43]. Furthermore, both of these materials are chemically inert, hence making both of these materials ideal for medical applications where reliability and biocompatibility are critical. Such properties provide long-lasting support and stability as they are able to withstand the stresses and forces applied to the body during the healing process [44].

Nonetheless, the problems with internal fixation, particularly with metal plates, are becoming more apparent as performance demands increases. One of the main issues is stress shielding: shielding of the bone from the normal stress or pressure it would otherwise experience. According to Wolff’s law, bones will adapt to the degree of mechanical loading. A plate with a higher stiffness and Young’s modulus will cause most of the weight to be supported by the implant while the bone only bears part of the load [43]. This change in how stress is distributed can lead to bone loss in the interface of bone and implant. Therefore, this can prevent secondary healing by stopping bone remodelling and callus formation [42].

There are several varieties of fixation plates used in orthopaedic surgery, each designed to serve a purpose and address specific types of fractures. The most common types include compression plates, neutralisation plates, buttress plates, tension band plates, bridging plates, and locking plates. These plates, along with their functions, are presented in Table 1.

**Table 1:** Overview of common orthopaedic fracture fixation plates and their functions Adapted from the Orthopaedic Trauma Association [45]

Plate	Function
Compression	Compression offers rigid fixation and absolute stability, making it particularly effective for transverse fractures where placing a lag screw is not feasible. It can be used either on its own or alongside lag screws: Alone: for transverse or short oblique fractures where lag screw placement is challenging. In combination: the plate is used to apply compression before placing the lag screws.
Neutralisation	Prevents lag screws from being subjected to shear, bending, or rotational forces at the fracture site. It can be used in combination with lag screws, whereby the lag screws provide compression, while the neutralisation plate helps preserve bony alignment and prevents the screws from failing by stabilising the fracture site against the forces acting on the bone.
Buttress	Counteracts vertical shear forces during axial loading and prevents sliding/shortening of fracture fragments under axial pressure. Plates can be used with or without lag screws to provide buttress.
Tension Band	Converts tensile (pulling) forces into compressive forces at a fracture site. It is particularly useful in fractures involving tension or avulsion, where the forces pulling apart the bone need to be counteracted, such as the patella and olecranon.
Bridging	Ideal for complex, comminuted fractures in the metaphysis or diaphysis, or when direct access to the fracture is challenging because of the soft tissues around it. The aim is to preserve fracture biology by avoiding direct disruption of the fracture zone and to achieve indirect reduction and relative stability, rather than achieving anatomic alignment and absolute stability.
Locking	A locking plate has threaded holes that allow the screws to "lock" into the plate itself, thus providing axial and angular stability. It is particularly useful in patients with compromised bone quality such as osteoporotic or weak bones. This is because the load is distributed across the entire construct.

Intramedullary nails

IMNs are widely regarded as the gold standard for the treatment of diaphyseal fractures in long bones as well as certain metaphyseal fractures. It acts as an internal splint, providing stabilisation for the fracture, and is commonly used for fractures of the tibia, femur, and humerus [46]. The primary objective of the IMN is not to achieve anatomical reduction but to restore the length, alignment, and rotation of the limb, facilitating functional recovery. The IMN offers relative stability rather than absolutely stability, meaning it allows for a certain degree of controlled motion at the fracture site. It functions primarily as an early load-sharing device, distributing mechanical forces across the fracture site, rather than a load-bearing device that takes on the full weight, and is particularly helpful in offloading comminuted fractures. Studies have shown that, in the initial stages of fracture healing, the IMN carries up to 60% of the load, thus providing critical stabilisation during the early phase. However, the overall mechanical stability of the construct improves as the fracture heals, and the majority of the load is transferred to the bone [47].

## Conclusions

The principles of fracture fixation are pivotal in guiding the healing process, with a strong emphasis on restoring anatomy, achieving stable fixation, maintaining blood supply, and enabling early mobilisation. The understanding of mechanical factors, particularly Perren’s strain theory, highlights the importance of managing the strain environment at the fracture site to facilitate either primary or secondary bone healing. The choice of fixation method, whether rigid or flexible, directly influences the healing mechanism, with rigid constructs promoting primary healing and flexible constructs encouraging secondary healing through controlled movement. Internal and external fixation methods, each with their specific advantages, contribute to fracture stability and alignment, ultimately leading to enhanced healing outcomes.
